# Flame Retardant Coatings: Additives, Binders, and Fillers

**DOI:** 10.3390/polym14142911

**Published:** 2022-07-17

**Authors:** Mohd Meer Saddiq Mohd Sabee, Zarina Itam, Salmia Beddu, Nazirul Mubin Zahari, Nur Liyana Mohd Kamal, Daud Mohamad, Norzeity Amalin Zulkepli, Mohamad Danial Shafiq, Zuratul Ain Abdul Hamid

**Affiliations:** 1 Emerging Polymer Group, School of Materials and Mineral Resources Engineering, Universiti Sains Malaysia, Engineering Campus, Nibong Tebal 14300, Pulau Pinang, Malaysia; meersaddiq@gmail.com (M.M.S.M.S.); zeityzulkepli@gmail.com (N.A.Z.); danialshafiq@usm.my (M.D.S.); 2 Department of Civil Engineering, College of Engineering, Universiti Tenaga Nasional, Kajang 43000, Selangor, Malaysia; salmia@uniten.edu.my (S.B.); nazirul@uniten.edu.my (N.M.Z.); yana_kamal@uniten.edu.my (N.L.M.K.); daud@uniten.edu.my (D.M.)

**Keywords:** additive, binder, coating, filler, flame retardant, intumescence

## Abstract

This review provides an intensive overview of flame retardant coating systems. The occurrence of flame due to thermal degradation of the polymer substrate as a result of overheating is one of the major concerns. Hence, coating is the best solution to this problem as it prevents the substrate from igniting the flame. In this review, the descriptions of several classifications of coating and their relation to thermal degradation and flammability were discussed. The details of flame retardants and flame retardant coatings in terms of principles, types, mechanisms, and properties were explained as well. This overview imparted the importance of intumescent flame retardant coatings in preventing the spread of flame via the formation of a multicellular charred layer. Thus, the intended intumescence can reduce the risk of flame from inherently flammable materials used to maintain a high standard of living.

## 1. Introduction

Coating is a layer of a material or substance in the form of liquid, gas, or solid that is applied onto the surface of an object, which is commonly referred as substrate [1]. Basically, coating may be applied for different purposes, such as decorative, functional, or both [2]. For instance, paints and lacquers are coatings that have the dual purpose of protecting the substrate and being decorative. Artists may use paints only for decoration, whereas the paints on large industrial pipes are used for function: corrosion prevention and identification (i.e., blue for process water, red for firefighting control) [2]. Other than that, functional coatings can be used to modify the surface properties of a substrate, such as adhesion, wettability, corrosion resistance or wear resistance, and surface roughness [3]. In semiconductor device fabrication, which uses a wafer substrate, the coating is applied to introduce completely new properties and functionalities without affecting the bulk substrate, such as magnetic response or electrical conductivity, and becomes an essential component of the final product [4,5].

In fact, a major consideration for most coating processes is that the coating must be applied at a controlled thickness. Various processes are used to achieve this control, ranging from a simple brush for painting a wall to very expensive machinery used in the electronics industry [6]. Another consideration is ‘non-all-over’ coatings in which control over where the coating is applied is required. Printing is one of the examples of non-all-over coating processes in which only a specific area on the substrate is coated [7]. Apart from that, a thin film of functional material is applied to a substrate, such as paper, fabric, foil, or sheet stock, in many industrial coating processes. If the substrate begins and ends the process wound up in a roll, the coating process is known as ‘roll-to-roll’ or ‘web-based’. When a roll of substrate is wound through the coating machine, it is referred as web [8].

In the development of coating technology, the function of the final coating materials and the surface properties of the substrate play vital roles in determining the coating efficiency and effectiveness, which are governed by the proper selection of base materials, solvent, and additives in the coating formulation [9,10,11,12]. The process starts with the targeted coating specialization and the type of substrates used to perform the coating process. The type of functional additives incorporated in the formulation determines the characters of the coating system [13]. These vital characters include overall viscosity, flowability and sprayability, dispersibility and homogeneity, and the optical properties of the coating itself, such as opacity and transparency [14,15]. Moreover, the accurate dosages and mixing procedure of coating additives are also key vital factors in establishing the functionality of the coating system [16,17].

**Figure 1:**
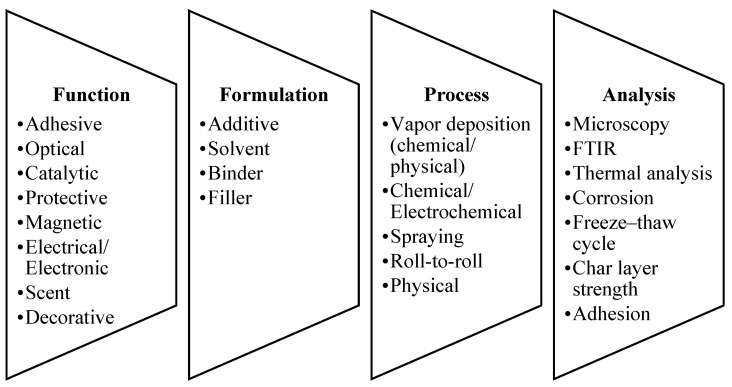
Coating details and categories based on function, formulation, process, and analysis.

The end properties and functionalization of the coating material also interplay with the interactions between the substituents in the formulation. The fundamental surface interactions and forces, such as van der Waals, hydrophobic, and ionic interactions, are the commonly discussed interactions in the coating system [18,19]. As a whole, each of the substituents added into the formulation will contribute to the net accumulation of these forces, in turn affecting the mentioned key properties of the coating materials and their ability to adhere to the substrate. Figure 1 shows the details of coatings based on various classifications.

Function: The implementation of coatings to achieve specific functions includes adhesive (tape, iron-on fabric, pressure/temperature-sensitive label, primer, polytetrafluoroethylene (PTFE)) [20], optical (mirror, UV-absorbent, tinted) [21], catalytic (self-cleaning glass), protective (waterproof, damp-proof, wear resistance, antifriction, antiscratch, barrier, anticorrosion, sealant, thermal insulation, fire protection, antimicrobial surface, antifouling, antigraffiti) [22,23,24], magnetic (cassette tape, floppy disk, mass transit ticket), electrical/electronic (magnet wire, resistor, conformal antenna), scent (scratch, sniff sticker), and decorative (reflection).

Formulation: Four elements are required in the formulation of coatings: additive, solvent (substance that dissolves a solute, resulting in a solution), binder, and filler. The details of these elements are discussed in the following subsections.

Process: Various approaches can be used in order to fabricate coating materials, including chemical vapor deposition (epitaxy, sherardizing, electrostatic spray) [25], physical vapor deposition (ion plating, magnetron sputtering, arc deposition, electron beam, laser deposition, vacuum deposition) [25,26], chemical/electrochemical (conversion coating, plasma electrolytic oxidation, phosphate coating, ion beam, electroplating, anodizing) [27], spraying (painting, high-velocity oxygen fuel, plasma spraying, thermal spraying, powder coating, kinetic metallization) [28,29,30], roll-to-roll (gap coating, gravure, hot melt, Meyer bar, silk screen, roller coating, extrusion, slot die, inkjet, lithography, flexography) [31], and physical (spin coating, dip coating, Langmuir–Blodgett) [32,33,34].

Analysis: Characterizations have to be performed to evaluate the coating performance for specific targeted applications: microscopy, Fourier-transform infrared (FTIR), thermal analysis (Bunsen burner, furnace, thermogravimetric analysis (TGA), limiting oxygen index (LOI)), corrosion, freeze–thaw cycle, char layer strength, and adhesion.

In fact, coating plays a significant role in many industrial applications especially for its protective functions. One of the major concerns is the occurrence of flame due to the thermal degradation of the polymer substrate as a result of overheat. Therefore, coating is the best solution to solve this issue as it functions to protect the substrate from igniting the flame. The following subtopics discuss in-depth flame retardant coatings.

## 2. Thermal Degradation and Flammability

The exposure of organic and inorganic compounds to heat causes their thermal degradation. Basically, a flame is formed when the thermal degradation of combustible materials is oxidative and is characterized by the generation and emission of heat and light [35]. The flame is the light emitted by the fire and serves as a visual indicator of the heat generated. Typically, combustion is a gas-phase phenomenon, where volatile combustible species oxidize exothermically [36]. On the other hand, afterglow combustion is a type of non-gas-phase combustion in which the substrate is oxidized in the condensed phase to produce both solid and gaseous products. It usually occurs at a temperature below the material’s ignition temperature. Meanwhile, in the solid phase, the carbon residue in a carbon-rich material is oxidized [37].

**Figure 2:**
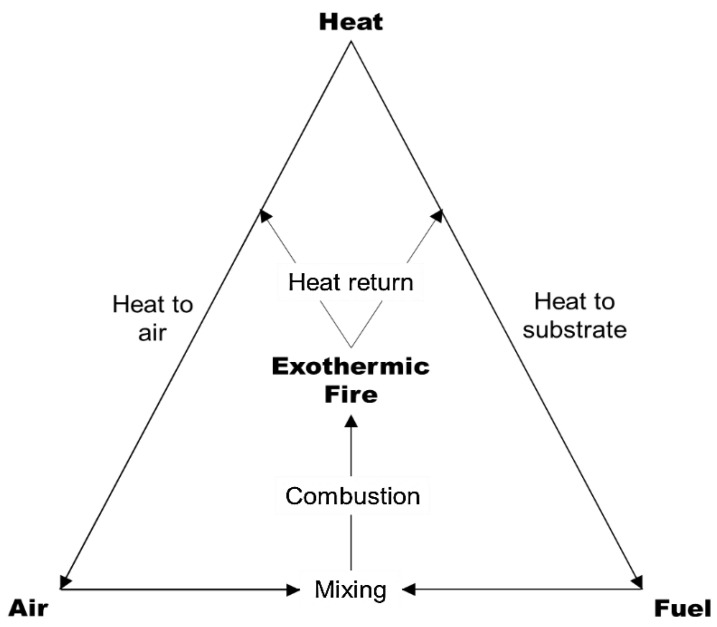
Emman’s fire triangle demonstrates the elements for a sustained flame.

In fact, three elements are required for a sustained flame to occur: fuel (consists of volatile combustibles derived from carbon-rich substances), heat (provided by the exothermic oxidative combustion of fuel), and oxidizing agent (oxygen supplied by air) [38,39]. Figure 2 illustrates that Emman’s fire triangle exhibits the three elements needed for a sustained flame.

The polymer substrate decomposes thermally, releasing smaller volatile compounds that act as fuel for the flame. These flammable species react with the oxygen in the air to form an ignitable mixture [40]. The volatiles undergo exothermic oxidation, and the material burns, which produces light and heat [41]. As shown in the fire triangle (Figure 2), the process becomes self-sustaining and operates with a feedback loop. The end result of the flame varies depending on the combustible compound. In the case of polymers, combustion gases primarily consist of carbon dioxide (CO_2_), carbon monoxide (CO), and water vapor (H_2_O). Meanwhile, the solid residue is primarily composed of carbon (C) and ash (oxidized metals) [42].

## 3. Flame Retardant

A flame retardant system is a compound or composition that is added to the polymer substrates in order to increase their resistance to combustion [43]. Excellent flame retardants must obstruct the supply of one or more of the elements required for long-term flammability. Basically, the purpose of flame retardants is to reduce the inherent flame risk of polymer substrates by lowering the rate of flame spread under fire conditions. The implementation of flame retardants can prevent a minor flame from escalating into a major crisis [44]. Therefore, to be effective, flame retardants must interfere with the polymer substrates’ degradation temperature. Normally, the most commonly used polymers as substrates degrade at temperatures ranging from 200 to 400 °C [45].

**Figure 3:**
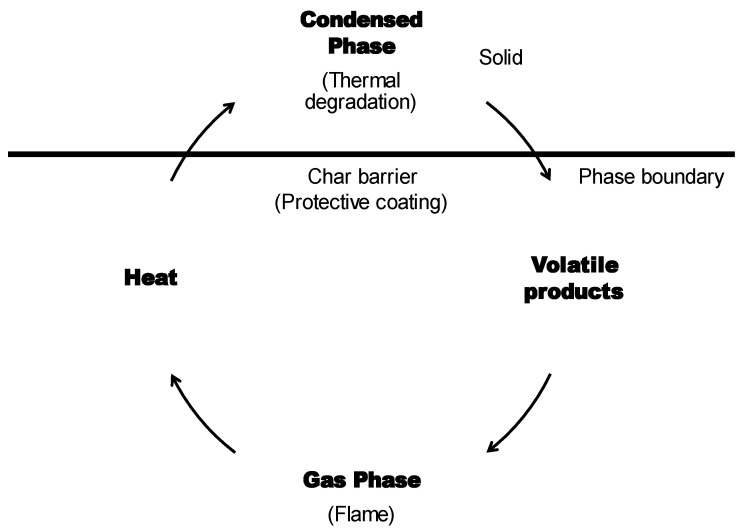
Flame retardant mechanisms.

However, it is well noted that a flame retardant is intended not to prevent the material from igniting, but rather to reduce the rate of flame spread and prevent sustained burning. This can be achieved by increasing the resistance of the polymer from ignition. In fact, ignition is unavoidable because most substances will ignite if exposed to high levels of flame stress–thermal radiation, which mostly occur due to the organic content of the polymer substrate [46]. Hence, the interference process of flame retardants with the polymers’ degradation temperature is required [47]. In fact, interference with the combustion process can occur in the gas and vapor (flame zone) phases as well as the condensed and solid phases (polymers melt), as illustrated in Figure 3 below [48].

A comprehensive and useful description of the mechanisms of a general retardant system is shown in Figure 3. There are several types of flame retardant systems, each with its own mechanism of operation. Basically, flame retardants disrupt the polymeric materials’ thermal decomposition pathway. Different compositions interact differently with different polymers, and thus, the use of flame retardants is very specific to the substrates for which they were designed [49].

Initially, the usage of halogen-containing flame retardants began to increase in the 1970s [50], while, the utilization of brominated systems increased dramatically in the 1980s [51]. Some halogen flame retardants that form acids during combustion work by inhibiting free radicals in the gas phase. When heated, they decompose into halogen radicals, which later obstruct the oxidation of volatile fuels. Subsequently, they react in the gas phase with oxygen, thereby reducing its concentration and extinguishing the flame [52]. In addition, synergists for halogen-based flame retardants include antimony-based and phosphorus-based compounds. These compounds aid in the scavenging of free radicals and the regeneration of halogen radicals [53,54].

The market began to shift from halogen-based to halogen-free flame retardants in the 1990s due to the fact that the systems emit toxic and corrosive gases, such as hydrobromic and hydrochloric acid fumes, as well as high levels of smoke [55,56]. Furthermore, halogen-containing retardants are difficult to dispose of, implying that the systems pose serious environmental risks [57]. The transition to more environmentally friendly flame retardants is gaining particular interest in the world, where the vast majority of current research is devoted to nonhalogen systems [58]. Alternative systems, on the other hand, are typically less effective and more expensive.

There are other types of flame retardants that emit a large amount of noncombustible gas, which able in diluting the amount of fuel or oxygen supplied to the flame. Basically, these types of flame retardants reduce flame spread via the formation of solid residues on the surface of the burning material. As a result, they can slow down the rate of heat release during combustion by modifying the heat transfer pathway to the polymer substrate [43,59]. Meanwhile, another class of flame retardants acts by forming a foam char on the combusting materials’ surface. These types of additives are commonly referred as intumescent systems [59]. Apart from that, this system can function by the combination of the mechanisms mentioned above.

There are several principles to explain the mechanisms of flame retardant systems. The terms and definitions describing the different principles of flame retardants are shown in Table 1 below.

**Figure 4:**
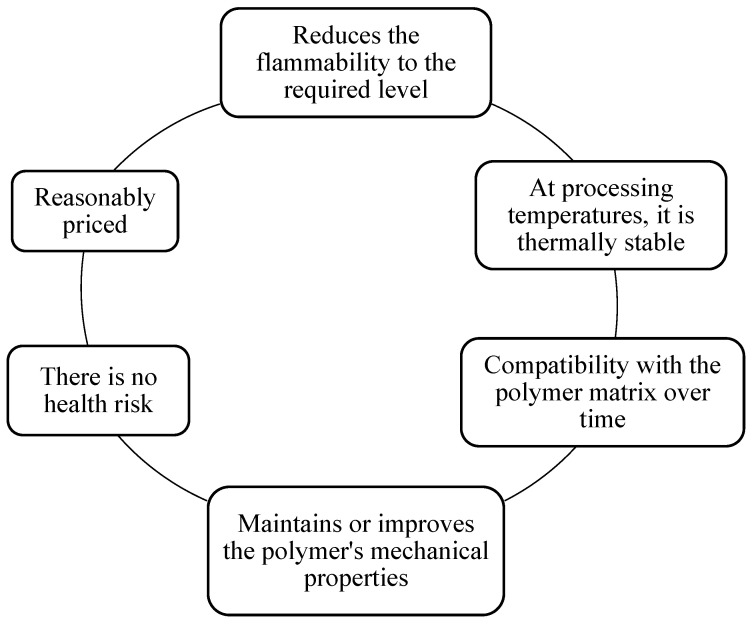
The properties of an ideal flame retardant system.

Another consideration of flame retardant systems is their effectiveness to overcome combustion issue. The requirements, such as low flame spread, low smoke emission, nontoxicity, and nonaltering performance of the substrate, are desirable for excellent flame retardant systems [67]. In addition, an ideal flame retardant has to fulfill the following properties, as illustrated in Figure 4 below [43,68,69].

In addition, flame retardants reduce the risk of flame from inherently flammable materials used to maintain a high standard of living [45]. Although bulk flame retardants have been proven effective for many years, there is now increased interest in the use of surface treatments to localize flame retardant chemistry at the exterior of a material, where combustion occurs, in order to preserve desirable bulk properties while minimizing the amount of additive required [70]. However, challenges remain to improve current flame retardants, as the success of these treatments is dependent on their scalability, durability, and ability to impart desired functionality while posing no environmental problems [71].

## 4. Flame Retardant Coatings

Flame retardant coatings (or spray) are noncombustible chemicals that are used in residential, commercial, and industrial buildings for a variety of reasons, including slowing the spread of a flame, reducing its intensity, and decreasing the amount of smoke produced [72,73]. As one of the well-established and most efficient methods, flame retardant coatings have been employed widely to protect a substrate against flame. Indeed, flame retardant coatings present several advantages: do not alter the intrinsic properties of the material (i.e., the mechanical properties), can be easily processed [45], and can be used on multiple substrates, such as metallic materials [74], polymers [75], textiles [76], and wood [77].

Insulation is a common method for protecting substrates from flame [78]. The ideal coating should have the characteristics of low thermal conductivity, nonflammability, great adhesion to the surface substrate, environmental durability, light weight, wear resistance, thinness, and low cost [79,80]. Currently, there are hundreds of commercially available coating materials for use on structural elements; however, none can cater to multifunctional properties required for ideal coating.

Insulating coatings are classified into three types, which are flame retardant polymers [46], thermal barriers [81], and intumescent coatings [82]. Flame retardant polymers are organic resins (i.e., brominated polymers) or inorganic materials (i.e., geopolymers) that are inherently flame resistant and commonly fabricated as thin film of less than 5 mm thickness, coated on the composite substrate [46]. Because of their high thermal stability and low thermal conductivity, these polymers are capable of delaying the ignition and flaming combustion of the substrate [43,46]. Meanwhile, thermal barrier coatings are typically made of ceramic-based materials that are nonflammable and exhibit low heat conductivity. Ceramic plasma-sprayed films (i.e., zirconia) and ceramic fibrous mats (i.e., silica, rock wool) are examples of these types of coatings [83]. Meanwhile, intumescent materials can protect against flame by undergoing a chemical reaction at high temperatures, causing the coating to foam and swell. Significantly, this reaction produces a highly porous and thick char coating with very low thermal conductivity, which protects composite materials from flame [84,85].

### Types of Flame Retardant Coatings

There are two types of flame retardant coatings, which are nonintumescent coatings and intumescent coatings. Nonintumescent coatings are essentially decorative and architectural, containing flame retardant additives designed to slow the spread of flame and smoke on combustible substrates [43,85]. They are further classified as class A, B, or C depending on their ability to contribute to flame and smoke. In fact, the rate of flame spread for this type of coatings is affected by both the substrate and the thickness of the film [86]. On the other hand, intumescent coatings basically swell under the influence of the heat to form a multicellular charred layer that acts as an insulator and slows the heat and mass transfer between the condensed and vapor phases [87]. This intumesced char can grow up to 50 times thicker than the original thickness of the applied coating [82,88]. In addition to that, there are pigmented/colored and clear/transparent varnishes of coatings available in the market. They are designed for use on different materials and react very differently when exposed to flame. They are also primarily used in the construction, transportation, wall and ceiling linings, and other areas that require products to fulfill specific requirements as an ideal flame retardant coating [73,89].

## 5. Intumescent Coatings

Intumescent coating protects against flame by the endothermic decomposition reaction process at high temperatures, causing the material to swell and foam into a highly porous, thick, and thermally stable char layer [90,91]. Because of the coating’s high void content and thickness, it can act as an insulation barrier to the underlying substrate against flame and heat [92]. Intumescent coating can be applied to structural elements by painting or spraying a liquid compound on them. Over a certain period of time, the compounds cure in the air to form a solid intumescent film [82,93]. Generally, the maximum coating thickness achievable with this method has to be less than 5 mm. If a thicker coating is required, it can be applied by directly bonding a fibrous intumescent mat to the substrate with high-temperature adhesive paste [78,94].

Basically, intumescent coatings impart flame protection via three reaction processes: (i) the coating material decomposes, (ii) inert gases evolved from the decomposition reaction are produced at a sufficient rate to channel back hot convective air currents, and most significantly, (iii) the coating expands into a highly porous char layer with a high resistance to heat conduction from the flame into the underlying composite substrate [95,96,97,98].

**Figure 5:**
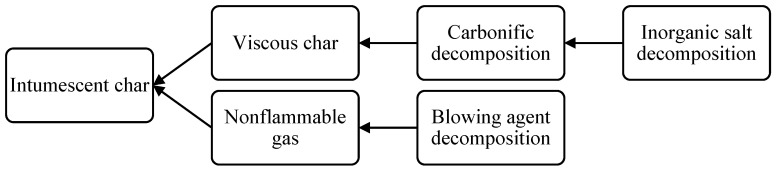
Schematic of intumescent reactions.

In fact, intumescent coatings are made up of a variety of compounds, each of which plays a specific role in the intumescence process. A carbon-rich (carbonific) compound, an inorganic acid or acid salt, an organic amine or amide, and a blowing agent are the four main types of compounds (spumific) involved in intumescent coatings [72]. In order for intumescence to occur, these compounds must go through a series of decomposition reactions and physical processes almost simultaneously, as well as in the correct order [93]. Figure 5 depicts the sequence of these processes, which contribute to the occurrence of intumescence. If the time between the processes is too long, or if they are not performed in the correct order, the coating will not intumesce [99].

Basically, the decomposition of the inorganic acid or acid salt within the coating initiates the intumescence process. The acid decomposition temperature must be sufficiently high that normal external heating (i.e., warming from direct sunlight) does not cause the coating to intumesce in the absence of flame; hence, it must be lower than the pyrolysis temperature of the composite substrate [56,87,100]. Furthermore, in order to ensure dehydration of the carbonific compound, the acid must decompose before any other compound in the coating. Linear high-molecular-weight ammonium polyphosphate (APP), zinc borate, organic esters, melamine (MEL) phosphate, and salts of ammonium, amide, or amine are among the commonly used acid compounds. These compounds decompose at temperatures ranging from 100 to 250 °C, which is lower than the pyrolysis temperature of most organic resins used in the composites. The usage of organic amides or amines can catalyze the acid decomposition reaction [72].

The carbonific is then decomposed via a dehydration reaction with the decomposed inorganic salts, converted into carbonaceous char in this reaction. The carbonific is a carbon-rich polyhydric compound that produces a lot of chars, which are usually a polycarbonate (starch or polyhydric alcohol) or phenol (phenol-formaldehyde) [101]. The hot and viscous char is then expanded by the blowing agent’s decomposition. The expansion of the char is dependent on the simultaneous decomposition of the carbonific and blowing agents; otherwise, the coating will fail to fully intumesce [102]. The blowing agent decomposes via an endothermic reaction that produces a large amount of nonflammable gases, causing the char melt to swell. The typically used blowing agents are nitrogen compounds, such as urea, guanidine, dicyandiamide, glycine, and MEL, which produce ammonia (NH_3_), carbon dioxide (CO_2_), and water (H_2_O) vapor [103]. Meanwhile, chlorinated paraffin is another efficient blowing agent that can be used, which produces hydrogen chloride, CO_2_, and H_2_O vapor. The gases congregate in small bubbles, causing the char to foam and swell. The coating eventually hardens into a thick multicellular material that slows the heat conduction from the flame into the composite substrate.

Subsequently, the thickness of an intumescent coating is increased many times from its original thickness when intumescence occurs. Excellent intumescent coating expands 50 to 200 times, forming a fine-scale multicellular network with cell sizes ranging from 20 to 50 µm and wall thicknesses ranging from 6 to 8 µm [82,93]. Oliwa et al. discovered that adding graphite flakes to an intumescent coating could increase flame resistance [104]. When heated, the flakes expand up to 100 times, resulting in a more effective insulating layer [105]. Other than that, the addition of inert fillers that aid in cell nucleation can be used to control cell size [106]. Fillers, such as titanium oxide (TiO_2_) and silica, are commonly used to reduce the average diameter of the cells. In fact, manufacturers closely control the exact chemical compositions of the compounds used in commercial intumescent coatings. While there is a wide range of compounds from which to formulate intumescent compositions, only a few are used in practice. The coating may also contain other additives for purposes other than intumescence, in addition to the compounds controlling the intumescent process. For instance, coatings may contain coalescing agents, thickeners, antioxidants, milled fibers, and coloring pigments for structural reinforcement [91,107].

In a flame, intumescent coatings are excellent heat insulators, which slow the rate of heat transfer into the substrate. Intumescent coatings can be extremely effective at delaying combustion, slowing heat release, suppressing flame spread, and lowering smoke density in the composite materials [85,108]. While intumescent coatings protect composite materials from heat and flame, they also have several drawbacks. Many commercial coating products have a weak bond to the substrate and frequently fall off during swelling, exposing the underlying composite directly to the flame [109]. Hence, when a coating is applied to vertical (i.e., walls) or overhead (i.e., ceiling) structures, this is a common occurrence. Therefore, to ensure adequate flame protection, the coating must be strongly bonded to the substrate and have sufficient mechanical strength [82]. Other issues with intumescent coatings include incompatibility with certain manufacturing processes, poor aesthetics, poor durability, rapid weathering (i.e., UV radiation, moisture absorption), and low resistance to wear and erosion [101,110,111].

In order to overcome these problems, the optimization of intumescent formulations based on a thermoset epoxy–amine system and the characterizations of their thermal performance have to be performed. For example, mineral acid (i.e., boric acid) and APP can be used as flame retardants, either separately or in combination, in intumescence fabrications [58,112]. It is discovered that using the formulation without flame retardants only provides properties similar to those of a virgin substrate. If boric acid or APP is added to the formulation separately, the flame protection performance is improved. However, the char does not adhere efficiently to the substrate and falls off, resulting in the substrate being exposed [113]. Meanwhile, the combination of these two flame retardant additives to the system yields the best properties and allows the char to adhere to the substrate. The improved behavior is attributed, in part, to the combination of phosphate (promotes substrate adhesion) and borate (produces a very hard char), resulting in the formation of boro-phosphate. Apart from that, the addition of boric acid to the formulation increases the viscosity by forming a hard glass (boron oxide), which traps gases and produces a char with high mechanical resistance [114].

## 6. Intumescent Flame Retardant Coatings

Tumid or tumescent means bulging or swollen, and intumescence is the process of becoming swollen. Certain substances swell when heated; this phenomenon is known as intumescence [97]. In an intumescent flame retardant, exposure to flame or heat initiates a series of physical and chemical processes, resulting in an expanded multicellular layer that acts as a thermal barrier, efficiently protecting the elements from a rapid increase in temperature and preventing the structures from collapsing under severe flame conditions [115]. This state is distinguished by the usage of flame-resistant insulating foam. Basically, the foam isolates heat and oxygen from the fuel source, effectively extinguishing the fire [116].

Intumescent flame retardant materials are essentially a special case of a condensed-phase mechanism. A complete description of intumescence necessitates evaluations of both the physical and chemical processes. Those mechanisms in this system are flame inhibition, heat loss due to melt flow and dripping, surface obstructions with char formation, acid-catalyzed dehydration, and char enhancement [117,118]. The formation of a charred layer basically affects the performance of intumescent flame retardants. Usually, flame retardant coatings are primarily based on ‘classical’ intumescent systems, where research in this field is extensive, and many formulation parameters can be adjusted and controlled. As a result, various intumescent formulations can be developed to achieve the specific flame protection requirements, which are firmly dependent on the substrate used and targeted applications [119].

### 6.1. Mechanisms of Intumescent Flame Retardant Coatings

#### 6.1.1. Physical Mechanisms

**Figure 6:**
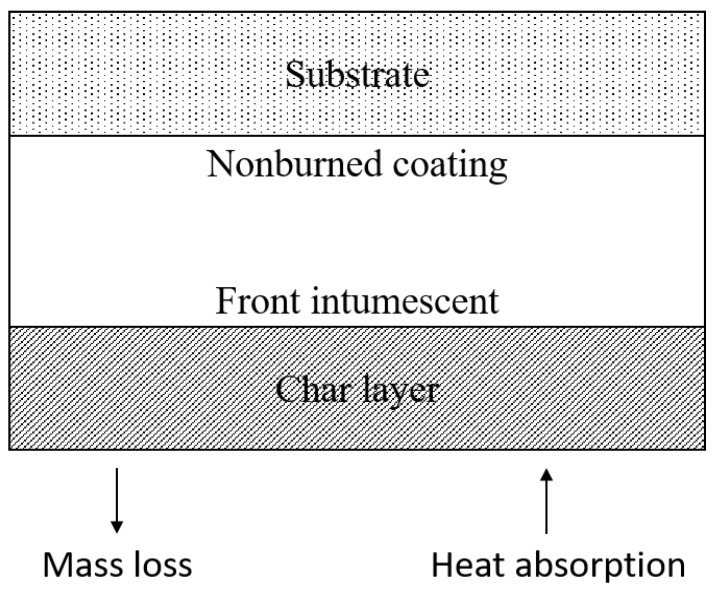
Schematic of multilayer formations during the flame process in intumescent flame retardant coatings.

Generally, the formulations for intumescent flame retardants include a phosphorous compound (i.e., APP), char-forming polyol (i.e., pentaerythritol (PER)), and blowing agent (i.e., MEL) [56,120,121]. In order to ensure that all compounds are in contact with each other, a binder is also required in the formulations [122]. Mostly, intumescent flame retardants are applied in paints and coatings [73]. With such intumescent coatings, the burning coating can be seen as a block made up of several separate layers, as illustrated in Figure 6. The substrate is represented by the upper layer, which is protected by the intumescent coating, which consists of nonburned coating and the char layer. The char layer is followed by the intumescent front, which is where foaming reactions occur. On the other hand, the nonburned coating layer does not contribute to that reaction as it still contains the flame retardant. Because char foam acts as a physical barrier to heat and mass transfer, it interferes with the combustion process [73,97,123].

As mentioned earlier, three elements are required for a mixture to be an efficient intumescent system: inorganic acid (dehydrating agent), blowing agent (spumific), and carbon-rich polyhydric material as a char former (carbonific) [124,125]. The ratios in which the various compounds are present are also a critical concern, where the best ratio has to be resolved experimentally. One or more of these substances could be substituted for others in the same class or group in which further research has revealed that incorporating two or more of the elements for intumescence into the same molecular complex results in more efficient intumescent systems.

The foamed char formed on the surface of the burning material contributes to the effectiveness of intumescent flame retardant systems. The char acts as a physical barrier to heat transfer to the combustible material’s surface. Other than that, the char layer prevents oxygen from reaching the site of combustion [126]. As shown in Figure 6, the formation of char slows the rate of temperature increase on the surface above the char layer. On the other hand, as carbonifics, halogenated compounds such as chlorinated paraffin and nitrogen-based compounds are normally used in intumescent coatings because of their environmental friendliness [127]. This is parallel to char-forming flame retardants, which have many advantages over other systems because they emit less smoke and toxic gases. Moreover, the smoke produced is less corrosive, and the scrap can be disposed of easier after usage [126,128].

#### 6.1.2. Chemical Mechanisms

**Figure 7:**
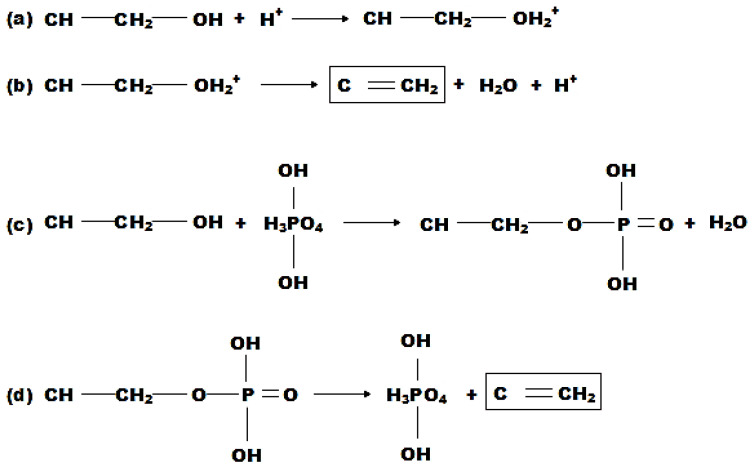
General mechanisms of intumescent flame retardant coatings.

Basically, simple acid-catalyzed and dehydration reactions are frequently described in the chemistry of intumescent flame retardant coating systems [46]. This is demonstrated by four reactions, as depicted in Figure 7. The first two reactions ((a) and (b)) demonstrate acid-catalyzed depolymerization. Meanwhile, (c) and (d) show the dehydration of the substrate in the presence of phosphoric acid [129,130]. Both (b) and (d) reactions produce C=CH_2_ fragments at the chain ends, yielding the same result (red square in Figure 7). Carbon-rich char residues are formed when these fragments condense. In brief, phosphorous compounds phosphorylate carbonifics (i.e., PER) to produce polyol phosphates. These polyol phosphates are then degraded and form the char layer [131].

In conclusion, the intumescent system’s protection mechanism is basically based on the formation of a charred layer that acts as a physical barrier, slowing heat and mass transfer between the gas and condensed phases [121,132]. When large amounts of thermally stable carbonaceous residue are heated, intumescent systems decompose and form [133]. The primary objective of intumescent materials is significantly improving the thermal protection, where heat transfer is limited by the formation of the intumescent shield. Swelling in intumescent systems is critical for firefighting capabilities; therefore, a basic understanding of the mechanisms that cause the expansion is essential [134]. Other than that, temperature gradients and heat transfer are also important factors that need to be considered in the intumescent behavior [135,136]. The effect of expanding bubbles on the temperature field, particularly, cannot be deteriorated. Additionally, a refined selection of components—char formers, carbonizing, dehydrating substances, and modifiers—is vital to make the intumescent flame retardant effective, allowing for a maximum degree of carbonization and, thus, an efficiency of the protective char [137].

## 7. Flame Retardant Coating Formulations and Designs: The Implementation of Polymer Materials

The widespread use of polymers in many applications has become a major concern in fire safety, which leads to a massive increase in the development of flame retardant polymer materials. New strategies and legislation regarding flame retardant polymers to save lives and property have also been rising, as well as flame retardancy science, economics, and technologies, which are also constantly evolving. Recent advances in the knowledge of flame retardant polymer materials, as well as their flame retardant properties, specifically address the progress made and the future prospects for designing precise structures using innovative technologies, particularly their flame retardancy performances. Indeed, the technologies of innovative flame retardant polymer materials are nearing a practical juncture in the near future. Normally, the formulations of intumescent flame retardant polymer materials consist of additives (Section 7.1), binders (Section 7.2), and fillers (Section 7.3), which are further discussed in the corresponding subtopics below.

### 7.1. Additives

Research on the development of flame retardant additives is critical. Many of the prepared flame retardants are problematic due to their negative effects on human health and the environment because the formulations are neither green nor sustainable because of relying on synthetic processes that use fossil fuels [138]. Therefore, in order to overcome the issues with synthetic derivatives for flame retardant coatings, developing more sustainable and nontoxic alternatives is significant. Many research groups have focused on developing new bio-based flame retardant additives for synthetic polymers, such as polysaccharides, proteins, lipid, chitosan, and microfibrillated cellulose.

Basically, additives are used to improve the flame performance of combustible products. In general, additives are substances added to a combustible material to delay or suppress ignition and reduce the rate of flame spread when exposed to flame impingement [139,140]. Numerous studies have been conducted on the properties of these materials as an additive for a wide range of polymers used. On the other hand, additives in flame retardant systems are basically not chemically attached to the surrounding system; however, additional research is being conducted to graft additional chemical groups onto these materials, allowing them to become integrated without losing their retardant efficiency [45,141]. Hence, it renders these materials nonemitting into the atmosphere when applied. In addition, most additives used in flame retardant systems have been approved by the U.S. Environmental Protection Agency (US EPA) due to their low environmental impact [142,143,144]. Below are some promising additives that can be used to formulate an effective intumescent flame retardant coating system.

#### 7.1.1. Ammonium Polyphosphate

Ammonium polyphosphate (APP) is an inorganic salt of ammonia (NH_3_) and polyphosphoric acid. This branched or unbranched polymeric compound’s chain length (*n*) varies, where *n* can be greater than 1000. Short linear-chain APPs (crystalline form I (APP I)) (*n* = 100) are more water sensitive (hydrolysis) and less thermally stable compared with longer branch-chain APPs (crystalline form II (APP II)) (*n* > 1000), which have very low water solubility (0.1 g/100 mL) [137,145]. APPs are known to be nonvolatile and stable compounds. At temperatures above 300 °C, long-chain APPs begin to decompose into polyphosphoric acid and NH_3_, whereas short-chain APPs begin to decompose at temperatures above 150 °C [146,147]. Therefore, it is crucial to match the crystalline form of the APP to the polymer decomposition temperature.

The incorporation of APP into oxygen or nitrogen-containing polymers (polyamides, polyesters, and polyurethane) is well known to cause polymer charring [60,148,149]. Thermal dehydration of APP basically produces free acidic hydroxyl groups (–OH), which are condensed to form a crosslinked ultraphosphate and a polyphosphoric acid with a highly crosslinked structure [117]. Polyphosphoric acid can react with oxygen or nitrogen-containing polymers, which catalyzes the dehydration reaction and leads to the formation of char [131,150]. The efficiency of APP is also determined by the level of its incorporation. APP is ineffective in aliphatic polyamides at low concentrations; however, at high concentrations (>10% in polyamide-6,6; >20% in polyamide-6,-10,-11,-12; and >30% in polyamide-6), it becomes very efficient [151].

#### 7.1.2. Pentaerythritol Phosphate Alcohol

Pentaerythritol has long been used in flame retardant formulations as a char former [152]. However, its beneficially low cost is offset by its unfavorably high level of water solubility. For the effective intumescent systems in flame retardant coating, the additives used have to be water insoluble, thermally stable, and nonhalogenated [153]. Therefore, the creation of phosphorus-rich pentaerythritol (pentaerythritol phosphate alcohol (PEPA)) derivatives can enable the success of an intumescent flame retardant specifically for polypropylene. In fact, carbonate and phosphonate ester derivatives of PEPA are promising components of an optimum polypropylene intumescent flame retardant.

When PEPA is used as both a carbon and an acid source, it has been discovered that intumescent flame retardant systems exhibit excellent char-forming ability and thermal stability [154,155,156]. For instance, the thermal decomposition behavior of a PEPA/melamine mixture can achieve a maximum fire retardancy effect and has a noticeable effect in polybutylene terephthalate (PBT) [157]. In addition, sulfur-containing caged phosphate ester in certain PEPA derivatives shows outrageous flame retardancy in polypropylene as well. Apart from that, hydroquinone has the ability to endow an active end-functional group of hydroxyls (–OH) that can participate in the formation of PEPA derivatives. The benzene groups in hydroquinone can significantly improve the charring agent’s carbon content [158]. As a result, increased carbon and phosphorus content directly decreases the stereo-hindrance effects that elevate the suppression of flame spread, which determines that intumescent flame retardant coating systems are competent [159,160].

#### 7.1.3. Melamine

Melamine (MEL)-based flame retardants are a small but rapidly expanding scale in the flame retardant market. These products have several benefits over a conventional flame retardant, including low toxicity and smoke density [161], cost-effectiveness [148], environmental safety [58], and corrosion resistance [148]. Hence, MEL offers excellent protection when used as an additive in the fabrication of flame retardant coatings, especially intumescence. There are three chemical groups that can be distinguished in this family of nonhalogenated MEL flame retardants: (i) completely pure MEL, (ii) MEL derivatives (salts with organic or inorganic acids, such as phosphoric, boric, cyanuric, pyro/polyphosphoric acid), and (iii) MEL homologues (melam, melem, melon) [162].

Because of their ability to employ various modes of flame retardant action, MEL-based flame retardants basically exhibit excellent flame retardant properties and versatility in use [163]. Generally, MEL-based flame retardants work by interfering with one of the three components that start or sustain combustion: heat, fuel, and oxygen [148,164]. In fact, MEL has the ability to interfere with the flame process at all stages to delay ignition by creating a heat sink via endothermic dissociation prior to endothermic sublimation of the MEL itself [165]. In addition, the subsequent decomposition of MEL vapors produces an additional larger heat sink effect [166].

Apart from that, MEL can play an important role in the formation of a char layer during the intumescent process as well. Char stability is improved by multiring structures formed during MEL self-condensation [167]. MEL can also act as a blowing agent for the char, improving the char layer’s heat barrier functionality [168]. Additionally, MEL-based flame retardants are currently used primarily in intumescent coatings, flexible polyurethane foams, polyamides, and thermoplastic polyurethanes [169]. The market for MEL-based flame retardants is believed to expand in the near future due to continued research and application development work in the direction of polyolefins and thermoplastic polyesters [170,171].

#### 7.1.4. Boric Acid/Borate

Boric acid is a chemical compound that occurs naturally and is composed of hydrogen, oxygen, and boron. It can be found naturally in almost all fruits and vegetables, as well as in some nuts and grains, and can also be mined from the ground [172,173]. Generally, boric acid is a nonhazardous substitute for chemical flame retardants that has been extensively studied and consistently deemed to have low toxicity for both animals and humans, and is listed as a noncarcinogen by the US EPA [174].

Basically, boric acid, also known as borate, acts as a flame retardant in a variety of ways, including preventing flame combustion, promoting char formation, and suppressing smoldering, glowing, and smoke [43,175]. In addition, boric acid releases water to help extinguish the flame; other than that, it has a char-forming value on the surface of the cellulose due to the presence of boron [176]. On the other hand, when neutralizing acid materials, boric acid acts as a buffer in which it can protect metals in cellulosic materials by acting as an anticorrosive [177], prevent combustion of flames [178], and promote the formation of char [179], and if combined with other flame retardants, it has a synergistic effect [180,181].

### 7.2. Binders

The binder in the intumescent flame retardant coatings is vital because it contributes to the formation of a uniform foam structure and the expansion of the char layer [182]. Binders, such as polyvinyl acetate emulsion, vinyl chloride latex, alkyd resin, and acrylic resin, are extensively used in the formulations of intumescent flame retardant coatings. These binders basically perform well in order to overcome flame; however, their chemical compositions usually emit a bulk of toxic gases and smoke when exposed to the flame [87,183]. Therefore, the usage of a water-based binder, such as epoxy emulsion, can be implemented to reduce the release of smoke and toxic fume while maintaining the quality and effectiveness of intumescent flame retardant coatings in flame protection [184]. Below are some potential binders that can be used in the fabrications of effective intumescent flame retardant coating systems.

#### 7.2.1. Ethylene Vinyl Acetate

Ethylene vinyl acetate (EVA) is a polar vinyl acetate comonomer containing ethylene that can be synthesized by varying vinyl acetate contents. It is widely used in numerous applications, where EVA varies greatly, depending on the vinyl acetate content of the copolymer [185,186]. However, the high flammability of EVA prevents it from maximizing its usage [187]. Therefore, the addition of flame retardant agents is necessary to improve the flame retardancy of EVA. It is also noteworthy that the development of flame retardant EVA compounds has to be halogen-free as this system releases toxic and corrosive gases and produces high levels of smoke, which leads to environmental and health risk [127,188].

Recently, in many applications, inorganic hydroxide fillers (sometimes layered double hydroxides) and intumescent flame retardants have been intensively studied and commonly used in the production of halogen-free flame retardant EVA copolymer systems, taking into account life and environmental safety concerns [153,189]. They decompose in both the condensed and gas phases, decreasing the temperature of the polymer and releasing water into the gas phases, which dilutes the flame. They also function as catalysts for the oxidation of carbonaceous residues, lowering the CO/CO_2_ ratio [190]. The oxides formed during the decomposition process can contribute to the formation of an insulative charred layer, which acts as additional protection for the polymer [87]. Moreover, there have been a lot of interests in intumescent flame retardant systems that can be used in EVA, which have proven to be very efficient due to high insulative properties, apart from improving the flame retardancy and smoke suppression performance, as well as convenience and cost-effectiveness [191,192,193].

#### 7.2.2. Epoxy Resin

Epoxy resin is an organic macromolecule that can be inter- and intramolecularly crosslinked to form a three-dimensional (3D) polymer network, making it the most adaptable type of thermoset polymer [194]. Generally, epoxy resin is used in a wide range of applications, ranging from general use to high-performing materials, such as adhesives and protective and decorative coatings [195], owing to their high versatility from a chemical and processing standpoint and ability to be tailored for particular required properties [196,197]. In fact, epoxy resin can be used for various types of materials, such as fabric, wood, glass, and metal in many household goods and structural, electronic and construction applications. However, most of the applications necessitate adherence to specific flame safety regulations.

In fact, the combustion of epoxy resin results in a high rate of heat and smoke release, which could have serious consequences [198,199]. Hence, it is critical to modify epoxy resin to improve flame retardancy without sacrificing its other properties that are required for its specific applications. With an increased emphasis on environmental protection, flame retardants for epoxy resin tend to be nontoxic, efficient, systematic, and multifunctional [67,200,201]. Basically, the addition of flame retardants to epoxy resin has numerous advantages, including simple processing [202], low cost [67], a diverse source of raw materials [203], and an obvious flame retardant effect [67]. Organophosphorus, silicon-containing compounds with an intumescent effect, nanocomposites, and metal-containing compounds are common flame retardants used for epoxy resin [204].

The currently reported flame retardants based on polymers have the ability to provide epoxy resin with flame retardancy, low flammability, heat resistance, heat release rate, and thermal stability, other than their nearly negligible negative effects on the mechanical properties or glass transition temperature of epoxy resin [205,206,207,208]. It has also been discovered that flame retardant systems for epoxy resin produce significantly more and stronger char with better uniformity and smaller average bubble size.

#### 7.2.3. Polyamide

Polyamide is an engineered thermoplastic with chemical, mechanical, molding, and electrical insulation properties [209]. One of the typical polyamides used in many industrial applications is polyamide-6,6 with many amide bonds in the main chain, allowing it to have good processing properties, low melt viscosity, excellent thermal and mechanical, strong chemical and electrical resistance, as well as fatigue and abrasion resistance [210,211]. However, polyamide-6,6 exhibits flammability and smoke-emitting issues, endangering people and the environment, thus limiting its widespread use in high-temperature fields [212].

Therefore, improving the flame retardancy of polyamide-6,6 is critical to enhance its flame retardancy. The addition of polyphenylene oxide to polyamide-6,6 fabrications can solve problems regarding burning and smoke releasing as this substance is an amorphous polymer with excellent thermal stability, mechanical strength, and flame retardancy due to its unique molecular structure [213]. In addition, it has been found that both additive type and reactive type of organic phosphorus flame retardants can improve the flame retardant performance of polyamide-6,6 [214].

On the other hand, it is well known that the inclusion of nanoclays (layered silicates) in another type of polyamide (polyamide-6) improves the flame performance, which is normally defined as a reduction in the peak heat release rate and an increase in the char formation [215,216]. Furthermore, when combined with conventional flame retardant additives, synergistic effects are observed, allowing for the possibility of reducing flame retardant concentrations in order to achieve a defined level of overall flame resistance [217]. Many of the previously cited references provide evidence that optimal flame performance occurs when the dispersion of both clay and flame retardant is maximized [215,218].

Apart from that, the emerging use of a caprolactam-based polyamide-6 matrix material for long-fiber reinforced automotive composites necessitates effective flammability reduction. Hence, flame retardants are required by stringent safety regulations to prevent the spread of flames, heat release, and the formation of toxic fumes, as well as to maintain the load-bearing capacity of the composite [68,219]. Commonly, flame retardants are added to this polymer matrix, which can affect the viscosity of the matrix, as a result, producing a compound that is capable of slowing the rate of heat transfer [52].

#### 7.2.4. Cellulose

Cellulose is an organic biocompatible and biodegradable polymer that is used in a variety of applications, such as medicine, water treatment, and food packaging [220]. Generally, cellulose nanocrystal (CNC) is fabricated via acid hydrolysis of cellulose fibers (i.e., hydrochloric, sulfuric, nitric, and phosphoric acids) [221]. Its unique optical and mechanical properties have piqued the interest of the global scientific community. Nevertheless, in certain applications that need high-temperature operations, cellulose discloses thermal degradation, which leads to heat generation [222].

There are two mechanisms that can ignite cellulose to form flame. Temperatures of 150 °C or higher can cause cellulose to thermally degrade into liquid, gaseous, tarry, and solid products. Volatile, flammable gases then ignite and provide additional heat to further pyrolyze the liquids and tars into more flammable vapors and to form residues, primarily carbonaceous char and a water–carbon dioxide gas mixture. This process is repeated until only the carbonaceous residues remain [223,224]. Meanwhile, the second pathway uses carbonaceous char from pyrolysis and operates at lower temperatures. The resulting char is oxidized in a slow and localized process known as glowing or smoldering combustion. Basically, smoldering combustion may occur in the charred area or consume the entire substrate, moving as a solid front rather than a flame in the gas phase [225].

In order to counteract this issue, boric acid, borax pentahydrate or borax decahydrate, ammonium phosphate, ammonium sulfate, aluminum trihydrate, aluminum sulfate, and gypsum can be used as chemical fire retardants for cellulose [226,227,228]. In most cases, these chemicals are used in combination with two or three different chemicals. To gain a better understanding of the subject of flame retardancy, it is necessary to comprehend the various flame retardant mechanisms demonstrated by the aforementioned flame retardants, a subtopic that is reliable for the vast majority of cellulosic applications. One of the mechanisms includes lowering heat loss to the surroundings by increasing thermal insulation to improve a substrate’s energy efficiency [229]. Additionally, because of its high insulating value, ease of installation, and low cost, cellulose loose-fill insulation is a popular insulating material [230,231].

### 7.3. Fillers

Particulate fillers can have a significant impact on the combustion properties of intumescent flame retardant coatings, such as their resistance to ignition and the extent and nature of smoke and toxic gas emission products [191,232]. This could be due to the dilution of the combustible fuel source, which slows the diffusion rate of oxygen and flammable pyrolysis products and changes the polymer’s melt rheology, affecting its tendency to drip [233]. In addition, depending on the fillers in terms of heat capacity, thermal conductivity, and emissivity of the flame retardant composition, it may also change, resulting in increases in heat transfer and thermal reflectivity effects that can also slow the rate of flaming [233,234].

Generally, fillers cannot be considered completely inert in terms of their effect on flame retardant combustion. However, some metal hydrates, hydroxides, and carbonates, in particular, can confer additional flame retardancy and smoke suppression qualities [43,235]. In this reaction, an endothermic decomposition occurs, which cools the solid or condensed phase and releases gases that dilute and cool flammable combustion products in the vapor phase. In addition to contributing to the overall smoke suppression, the inorganic residue remaining after filler decomposition may be highly significant in providing a thermally insulating barrier between the underlying polymer substrate and the external heat source [112].

**Figure 8:**
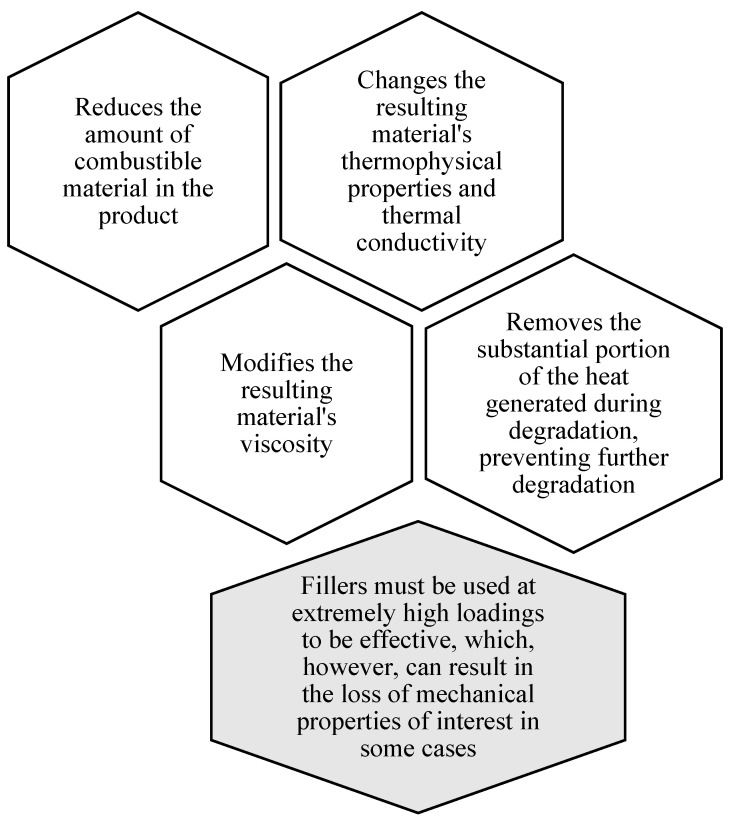
Objective of inorganic fillers in flame retardant coatings [87,240,241,242,243,244,245,246,247].

In addition to flame retardant effectiveness, these fillers should be ideally nontoxic, reasonably priced, free of conductive contaminants, and readily available in order to be commercially used [43,236,237]. To achieve the optimum flame retardant effect, thermal decomposition should occur close to the beginning of flame retardant degradation, with a subsequent release of flammable volatiles. Other than that, the shape and size of the fillers are also crucial factors to be considered [238,239]. Fillers’ particle size and the requirement for high addition levels to confer adequate flame retardancy are usually limiting their potential in intumescent flame retardant coatings, in terms of both processability and final physical properties [112]. Moreover, the usage of an inorganic filler can influence the reaction of intumescent flame retardant coatings to flame for several considerations, as further described in Figure 8.

All of these actions have an indirect impact on the flame performance of intumescent flame retardant coatings. Nonetheless, due to their high-temperature behavior, some minerals are more specifically used as flame retardant fillers. Metal hydroxides (i.e., aluminum, magnesium) and hydroxycarbonates are the most commonly used minerals as fillers in flame retardant coatings [101]. Aside from the effects mentioned above, these inorganic fillers also have a direct physical flame retardant action. These fillers are able to decompose endothermically as the temperature rises, thus absorbing energy [248]. Furthermore, they emit nonflammable molecules (i.e., H_2_O and CO_2_) that dilute combustible gases and can directly promote the formation of a protective ceramic or vitreous layer [101,249]. Below are some well-established fillers that can be used in the formulations of optimum intumescent flame retardant coating systems.

#### 7.3.1. Aluminum Trihydroxide (Al(OH)_3_)

Aluminum trihydroxide (Al(OH)_3_) is the most commonly used inorganic hydroxide as a flame retardant filler. Depending on the particle size, Al(OH)_3_ is processed at temperatures below its decomposition point, ranging from 190 to 230 °C [250]. Usually, Al(OH)_3_ is used as a flame retardant filler in thermoplastics, thermosetting resins, and elastomers that are processed at temperatures less than 200 °C with particle sizes larger than 50 µm [251,252]. It can be redissolved and precipitated to produce purer grades of Al(OH)_3_ that having smaller particle sizes. However, improvements to this process result in lower levels of iron, silica, and residual solid impurities [253]. Therefore, surface treatment needs to be performed in order to improve one or more specific mechanical properties of Al(OH)_3_ to be an effective flame retardant filler.

Usually, fatty acids or metal stearates are used for surface treatments on Al(OH)_3_ to limit additive aggregate and increase the elongation at break property [254]. Other than that, silane-based surface treatments are available with reactive (amino, vinyl, epoxy, and methacryl) and unreactive (alkyl group) substituents, which determine the incorporation of Al(OH)_3_ for tailored applications [249,255]. Meanwhile, other surface treatment options use phosphorus, titanium, and zirconium as the central element [256]. Regardless, titanates and zirconates are more expensive compared with silane-based surface treatments and can only be implemented for a very specific application [257].

#### 7.3.2. Magnesium Carbonate (MgCO_3_)

Inorganic chemicals, particularly magnesium compounds, are gaining popularity as common flame retardant fillers because of their effectiveness, low cost, and environmental friendliness, which have been used to treat polymers for flame retardancy [60]. Specifically, magnesium carbonate (MgCO_3_) has piqued the interest of researchers as an endothermic flame retardant that is stable enough to be incorporated into thermoplastics (i.e., polypropylene) without decomposition [45,258]. Basically, MgCO_3_ has a thermal stability intermediate between aluminum trihydroxide and magnesium hydroxide. At high filler concentrations (about 60%), MgCO_3_ is most effective, where it imparts a limiting oxygen index (LOI) of 28.2, which is more efficient compared with the flammability ratings for aluminum trihydroxide and magnesium hydroxide [259,260].

During the predecomposition stage (temperatures less than 250 °C), MgCO_3_ in the treated substrate absorbs heat, slowing its pyrolysis combustion and charring process. The release of water vapor and CO_2_ from MgCO_3_ during the combustion and charring stages results in the formation of a loose char layer, whereas the inner substrate is further charred during the char calcination stage, resulting in an increased char yield [261,262].

Generally, MgCO_3_ is able to emit inflammable gas and moisture to dilute the combustible gas in the flaming zone [141]. Simultaneously, the chemicals are converted to magnesium oxide prior to 500 °C, which has sufficient protective wall effects on the substrate [263,264]. In addition, the large endothermic loss of water during hydration, which also dilutes the combustion gases, is attributed to the effectiveness of MgCO_3_ [265]. Furthermore, the formation of an intumescent char on the burning surface aids this action, which eventually extinguishes the flame.

#### 7.3.3. Magnesium Hydroxide (Mg(OH)_2_)

Magnesium hydroxide (Mg(OH)_2_) is an inorganic flame retardant filler that is more thermally stable (temperatures above 300 °C) and is used in a variety of elastomers and resins, including engineering plastics and other high-temperature resins [256,266,267]. Mg(OH)_2_ is created through a variety of processes from magnesium-containing ores, such as magnesite, serpentinite, and dolomite, as well as brine and seawater. Some ores, such as brucite, hydromagnesite, and huntite, can be used directly as flame retardant filler or be converted first into Mg(OH)_2_ prior to use in the formulations of a flame retardant [268]. Mg(OH)_2_ used as a flame retardant filler typically has high purity (>98.5%). There are three available processes used to gain high purity of Mg(OH)_2_: the seawater and brine process, the Aman process, and the Magnifin process [269].

In general, the majority of Mg(OH)_2_ flame retardant grades are white powders with median particle sizes ranging from 0.5 to 5 µm [270]. Meanwhile, depending on the particle shape and size, the surface area of Mg(OH)_2_ ranges from 7 to 15 m^2^/g [271]. In addition, Mg(OH)_2_ is used at high loading levels, typically between 50% and 70%, to maximize its action of flame retardant [272,273]. However, in some cases, a small amount of Mg(OH)_2_ is used as a flame retardant filler due to the higher price of Mg(OH)_2_ in comparison with precipitated grades of Al(OH)_3_.

#### 7.3.4. Titanium Dioxide (TiO_2_)

Mineral fillers in intumescent flame retardant coatings, such as titanium dioxide (TiO_2_) (a pigment additive), have sparked widespread concern in recent years, which are commonly used in the coating industry. Basically, the incorporation of TiO_2_ to ammonium polyphosphate–melamine–pentaerythritol (APP–MEL–PER) systems improves the charring process in polymer matrices undergoing combustion [274,275]. The porosity and large expansion of the volume by the addition of TiO_2_ increase the flame retardancy effects: insulation of flame-proofing coating and height expansion [276]. Moreover, when used as a flame retardant filler in suitable intumescent formulations, TiO_2_ can improve the thermal performance properties and have a significant effect on porous and continuous char formation [238,277]. TiO_2_ also can enhance the antioxidation of the char layers and increase the coating residue weights, as well as improve the char residue’s foam structure, resulting in a decrease in the flame-spread rate [278].

Other than that, in flame retardant coatings, TiO_2_ can be used to prepare paints, revealing the product’s excellent antipollution and flame resistance [277]. It has been discovered that TiO_2_ can significantly improve the expandability and fire resistance of intumescent flame-proof coatings by forming a stronger, more char-forming layer with greater expansion and porosity [279]. When exposed to heat, the intumescent coating produces insulating foam and high expansions, acting as an effective barrier in the heat conduction into the substrate, therefore improving its thermal performance and char formation [91]. In addition to that, the combination of TiO_2_ with the natural anticorrosion agent (i.e., zinc borate) can improve the corrosion resistance, apart from the flame resistance performance of intumescent coating formulations [101,249].

#### 7.3.5. Expandable Graphite

Expandable graphite is a material that has exceptional thermophysical and mechanical properties. Furthermore, expandable graphite is a low-density carbon material with a unique set of properties, including a developed specific surface, binder-free pressing capacity, resistance to aggressive media, and low thermal conductivity, making it a promising material for both research and industrial applications [105,280]. One of the most common uses for expandable graphite is as a flame retardant filler. The heat of a flame basically causes graphite to expand and form an intumescent layer on the material’s surface. This can slow the spread of flame and reduce the harmful effect caused by the flame, which is the production of toxic gases and fumes [281,282].

It has been demonstrated that expandable graphite is an efficient additive that functions as both a blowing agent and carbonization agents, which has been shown to improve flame retardancy properties in a variety of substrates, such as polyurethane foam [282]. Highly thermostable graphite normally expands at temperatures ranging from 280 from 438 °C, resulting in the formation of a protected porous physical barrier between the substrate and the flame [105,283]. Furthermore, during the char formation process, void spaces within the char are formed, allowing airflow and cooling the flame environment (atmosphere), as a result, increasing the time to ignition of protected material [105].

Meanwhile, as a blowing agent, expandable graphite expands up to 100 times its original thickness [284]. In fact, this thickness of an isolative layer is greater than that of many intumescent agents used currently. In comparison with other intumescent flame retardants, the graphite-based char layer formed from an expandable flake can retain superior heat resistance [105]. Furthermore, a graphite flake is the only intumescent that expands with sufficient heat, allowing it to be used in rigidized systems.

Generally, the actual cause of an expandable graphite’s expansion/exfoliation is an increase in volume and pressure caused by the intercalant’s rapid heating. Modelling the intercalant as a liquid or solid phase that is fixed between the graphene layers can simplify the process of rapid heating [285]. Basically, this rapid heating of the treated graphite leads to the transition of the intercalant from a liquid or solid phase to a gas phase in which the volume of the intercalant increases about 100 times. Meanwhile, the pressure created by the increased volume causes the adjacent graphite layers to separate. The increment of these graphene layer spacings enhances the protection process of the substrate [105].

#### 7.3.6. Fly Ash

Fly ash normally contains a high concentration of soluble salts (i.e., sodium chloride (NaCl), potassium chloride (KCl)) and leachable heavy metals (i.e., cadmium (Cd), lead (Pb), zinc (Zn)), which classify it as a hazardous waste [286,287,288]. Therefore, pretreatments are required to reuse these ashes. The most common application of fly ash reuse is in building construction, where it is used as cement and concrete fillers [289]. According to the literature, fly ash can be used as a heavy metal adsorbent, micropollutant adsorbent, and material for CO_2_ sequestration [290]. In fact, the reuse of fly ash has several benefits, including the use of waste as a secondary raw material for achieving circularity in anthropogenic material cycles, the conservation of natural resources, and the reduction of waste landfilling [291,292,293].

Besides, it has been reported that using fly ash in polymer composites (i.e., polycarbonate or polyurethane, epoxy) can improve their thermal stability and oxidation resistance, as well as reduce the amount of smoke produced during combustion [294]. Other than that, fly ash can be filled into polymer foams to enhance the flame retardant properties of the foam materials, such as styrene and urethane foams [295]. Basically, fly ash is inert to flame, which does not emit toxic gases or smoke, which is crucial for environmental issues, and its use has the potential to save money by reusing waste materials as mentioned previously [296]. Apart from that, the use of fly ash is considered a nontoxic flame retardant filler for flame-proofing polymeric composite materials, as it has shown no harmful effect during its reaction [297].

On the other hand, the usage of stabilized fly ash as a flame retardant filler is also a promising alternative to traditional flame retardants (phosphorous and brominated flame retardants). The stabilized fly ash has been shown to have self-extinguishing properties similar to calcite [298]. Beneficially, unlike calcite, stabilized fly ash can be used in conjunction with other flame retardants to improve a material’s self-extinguishing performance due to synergistic effects [293].

Meanwhile, for other applications, fly ash carbonation has the ability to improve the mechanical and flame-proofing properties of silicone rubber composites. The addition of carbonated fly ash to silicone rubber increases the composites’ penetration time (26 times) as compared with neat cured silicone rubber [296]. Meanwhile, this carbonated fly ash exhibits up to 3 times higher penetration when compared with silicone rubber composites filled with other fillers, such as aluminum trihydroxide and magnesium hydroxide, which are commonly used fillers [299]. The carbonation process of fly ash also improves the mechanical properties of the fly ash–silicone rubber composite as well, contributing to an increase in flame retardant protection [300,301].

#### 7.3.7. Cenospheres

Cenospheres are low-density and hollow microspheres that are derived from coal-fired power plant fly ash waste [302]. The major components of the cenospheres are SiO_2_, Al_2_O_3_, and Fe_2_O_3_ [303]. Currently, efforts are being made to use cenospheres in value-added products, such as to reduce weight and increase strength in resins, concrete, plastics, paints, coatings, and ceramics [304]. Owing to the low density, high strength, astonishing thermal and electric capacity, excellent dispersibility and thermal insulation, high filling ability, and low coziness and viscosity of cenospheres, they have been used as fillers in various polymers, such as polypropylene, polyethylene, polyamides, and vinyl esters [299,303,305]. Basically, improvements in the thermal properties of cenosphere-containing composites are widely reported, which confirms the vitreous nature of cenospheres in terms of their high thermal stability and melting range (1350–1450 °C) [306,307].

Additionally, a substrate’s fraction increases with increasing temperature and carbon conversion ratio, but decreases with the addition of larger cenosphere particle sizes. The high fraction basically offers a plausible explanation for the extinction event at the late flame stage. Hence, the usage of a smaller size of cenospheres is more promising as a higher thermal stability is achieved to overcome the flame [308]. Other than that, the maximum flame length gradually decreases as the carbamide/cenosphere composite suppressant content increases, and the explosion suppression effect gradually improves With an additional amount of 40 wt% carbamide/cenosphere composite suppressant, a nearly complete explosion suppression of coal could be achieved. Therefore, the micro/nano multiscale complementary effect and deceleration–depressurization coupling effect were concluded as carbamide/cenosphere composite suppressant suppression mechanisms [302,309].

Apart from that, ceramic materials can be obtained using cenospheres, including iron-coated spheres [310]. In fact, ceramization is currently regarded as the next step in the development of the physical methods of protecting polymeric materials from flame. When exposed to high temperatures or flame, ceramization produces a rigid, durable ceramic coating on the surface of a polymer substrate. This ceramic layer formed on the material’s surface effectively limits the spread of flames on the surface and blocks oxygen diffusion into the inner layers of the substrate, as a result, reducing the amount of polymer thermal destruction products formed [255,299].

## 8. Conclusions

This review provides an understanding of the substrate materials’ thermal degradation that leads to the occurrence of flame. It is vital to determine the most suitable substances to be used for flame retardant systems. The detailed principles and mechanisms of the chosen intumescent flame retardant systems for specific applications are also crucial and must be acknowledged intensively so that successful implementation can be achieved. Besides that, the selection of additives, binders, and fillers is notably important for the intumescence to prevent the spread of flame significantly. Theoretical and experimental analyses of these materials have to be evaluated prior to applying them in the formulations of flame retardant coatings to ensure the success of systems during applications. This review found that intumescent flame retardant coatings can reduce the risk of flame from inherently flammable materials via the formation of a multicellular charred layer that functions as a thermal barrier, which is able to significantly prevent the flame from spreading.

## Figures and Tables

**Table 1:** The principles of flame retardant systems consist of inert gas dilution [60], thermal quenching [61], physical dilution [62], chemical interaction [63,64], and protective char [65,66].

Inert Gas Dilution	Thermal Quenching	Physical Dilution	Chemical Interaction	Protective Char
The thermal decomposition of the additive produces a large amount of inert and noncombustible gases. The oxygen and combustible species concentrations are reduced, and the flame is extinguished.	The endothermic degradation of the additive reduces or maintains the surface temperature of the polymer. Because the substrate temperature is lower, low combustible products are produced, and thermal degradation is detained.	A large amount of inorganic filler is mixed into the polymer matrix. As a result, the amount of flammable material is reduced, and the substrate’s flame resistance is increased.	Some flame retardants thermally dissociate into radical species, which then interfere with combustible gas-phase combustion.	As a result of thermal decomposition, the additive forms an insulating char barrier on the polymer’s surface. This char slows combustion by reducing heat transfer to the polymer, oxygen diffusion to the area of decomposition, and combustible diffusion to the flame zone.

## Data Availability

Not applicable.
